# Characterization of Bioadsorbents from Organic Municipal Waste

**DOI:** 10.3390/ma17091954

**Published:** 2024-04-23

**Authors:** Marcelina Sołtysik, Izabela Majchrzak-Kucęba, Dariusz Wawrzyńczak

**Affiliations:** Department of Advanced Energy Technologies, Faculty of Infrastructure and Environment, Czestochowa University of Technology, Dabrowskiego Street 73, 42-201 Czestochowa, Poland; izabela.majchrzak-kuceba@pcz.pl (I.M.-K.); dariusz.wawrzynczak@pcz.pl (D.W.)

**Keywords:** bioadsorbent, activated carbon, biocarbon, biowaste, biomass, chemical activation, waste management, circular economy

## Abstract

This article describes the production of bioadsorbents coming from seven different kinds of organic waste, produced in huge quantities in households, in a two-stage process. In order to determine the influence of the process parameters of carbonization (I stage) and activation with potassium hydroxide solution (II stage), the following analysis of the physicochemical properties of each sample at each stage processing was performed: base elemental composition, structure properties, surface morphology, thermal stability, crystallinity, and transmittance spectra characteristic bands. There was a lack of research on samples after each stage of waste processing in the literature. Addressing this allowed us to evaluate the transformative potential of each kind of organic waste included in the research and select the best waste for the production of bioadsorbents commonly used in environmental protection. Moreover, the results were compared with the ones in the literature. The utilization of particular kinds of organic waste seems to be especially important taking into account the strategy of waste management and sustainable development.

## 1. Introduction

**Figure 1:**
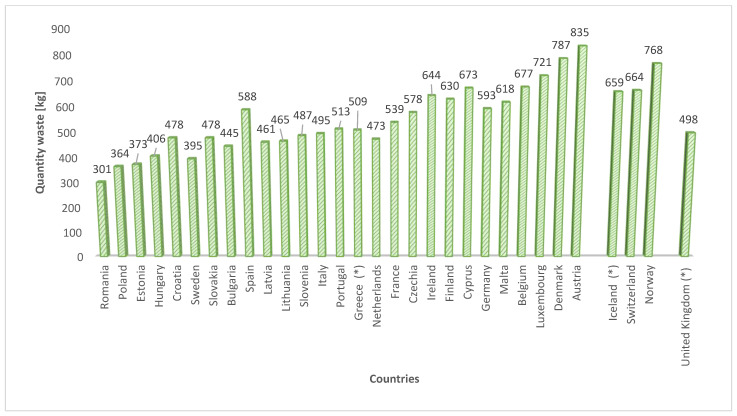
Municipal waste produced in the EU countries in kg per capita in 2022. * countries not belonging to the EU: Iceland and Norway (EEA countries) + Switzerland.

The quantity of municipal waste produced is on the rise, presenting a significant global challenge. In 2022, Poland recorded 355 kg of garbage per capita, which means a decrease of 5 kg compared to the previous year. In Europe, on average, there is 513 kg of municipal waste per capita, based on Eurostat data for 2022 (data for 2023 is not yet available). Figure 1 provides a summary of per capita waste collection in European Union countries in 2022 [1]. Only 40% of the municipal waste is selectively collected, leaving the rest untreated in landfills. The European Union is firmly committed to a circular economy in its economic policy. This concept, prominently featured in EU waste documents, prioritizes the reuse of waste materials and their reintegration into circulation. The Bureau of International Recycling designates recyclables as the “seventh resource,” recognizing recycling material as one of the planet’s six essential resources, alongside water, air, oil, natural gas, coal, and ores [2]. According to Directive (EU) 2018/851, the goal is to recycle as much as 65% of waste by 2035, a target that is currently distant [3]. In Poland, the proposed European directive aligns with the draft of the National Waste Management Plan 2028 (KPGO), adopting the same requirements as the EU directive [4].

Disposing of municipal waste in landfills presents numerous drawbacks. These drawbacks encompass extensive spaces designated solely for handling substantial waste quantities, challenges in waste transportation and collection, potential environmental contamination, and the risk of groundwater and nearby water reservoir pollution. Additionally, landfill sites create conditions conducive to the growth of pathogenic microorganisms, the presence of rodents, and the potential transmission of pathogens by birds. Moreover, landfills contribute to the emission of CO_2_ during the waste decomposition process [5].

In addition, 27% of total waste is organic waste [6], which is why there is a growing interest in its utilization. One of the straightforward methods for managing organic waste involves its use as a natural fertilizer for reclaiming degraded soils. Some waste has also been repurposed as raw material for the manufacture of recycled items such as cups and pots. Additionally, it has found application in cosmetics production.

In Poland, over 13 million tons of municipal waste are generated annually [7]. The largest concentration of waste is located mainly in the southwestern part of Poland, covering an area of several thousand hectares. Approximately 1.7 billion tons of waste have already been deposited in landfills, of which approximately 43% concerns only the Silesian Voivodeship. Currently, the costs related to transport and storage amount to several billion dollars nationwide [5]. Waste processing is a key challenge today.

In Poland, the yearly production of waste includes 120,000 tons of coffee grounds and 103,000 tons of tea grounds [8,9]. Each Polish individual consumes approximately 36 kg of potatoes annually, resulting in peelings that contribute to about 24,000 tons of waste [10].

Kitchen waste is a great compost material. It can be used to enrich the soil, improve soil structure, and support the growth of plants in gardens or for agricultural purposes. Recycling kitchen waste into fertilizers reduces the need for synthetic fertilizers, contributing to sustainable agriculture. Organic waste, including coffee and tea grounds, can be utilized for bioenergy production through anaerobic digestion or biomass combustion. The fibers in coffee grounds can be extracted and combined with other materials to create clothing, accessories, or even biodegradable packaging. By finding innovative ways to repurpose and recycle organic waste from coffee and tea grounds and other kitchen scraps, communities can contribute to a more sustainable and circular approach to waste management.

Various organic waste can be employed to create bioadsorbents, encompassing agricultural residues like rice husks, coconut shells, and sugarcane bagasse, in addition to food and industrial waste. These waste types contain cellulose, lignin, and other complex compounds suitable as raw materials for bioadsorbent production. Several examples of bioadsorbents derived from organic waste are summarized in Table 1. 

Researchers primarily focus on the investigation of bioadsorbents, neglecting the comparison of their properties before modification. In the two-step process of acquiring bioadsorbents, it is crucial to assess their characteristics both prior to and after modification. This evaluation provides insights into various aspects, including the effectiveness of the modification procedure. However, there is an absence of such information in the existing literature, with bioadsorbents being characterized solely after undergoing modification. In addition, articles frequently narrow their focus to a specific type of waste, creating difficulties in comparing the properties of waste generated under similar conditions.

This article delivers a thorough analysis of waste materials, specifically bioadsorbents. The characterization was conducted at two key stages: after the carbonization phase and following the activation stage. The selected organic waste, commonly found in Central and Eastern Europe, encompassed coffee and tea waste, potato peelings, and both green and brown walnut shells. Notably, this study marked the inaugural utilization of green coffee waste and green walnut shells for the production of bioadsorbents.

## 2. Materials and Methods

### 2.1. Materials

The organic waste examined in this research was heavily roasted coffee residue, regular roasted coffee residue, potato peelings, tea residue, green walnut shells, and green coffee residue; this waste was chosen for its widespread presence and accessibility in Central and Eastern Europe. In order to maintain the purity of the selected materials for research purposes, they were obtained directly and separately after they were produced (not recovered from biowaste). Table 2 provides the labels assigned to each type of organic waste, as well as their designations after the carbonization and activation stages.

For the bioadsorbent preparation, two varieties of the widely consumed dark roasted coffee were employed. 

The distinction between these two varieties arose from the distinct roasting processes they underwent: (1) HRoC, which underwent a dark roasting process conducted at temperatures ranging from 220 to 225 °C, possibly reaching 250 °C. This process led to dark brown or, in extreme cases, black beans, almost on the brink of charring. The high temperature in dark roasting tends to diminish caffeine and acidity. (2) RReC, which underwent light roasting at approximately 200 °C, resulting in a coffee with natural characteristics, high acidity, and elevated caffeine content.

Potato peelings (PP) were another material employed in the creation of biocarbons. Potatoes, a widely known plant since the 19th century in Europe and now globally recognized, come in numerous varieties, with Chilean varieties being particularly popular. The potato tuber consists of an inner tissue, typically yellow, enveloped by an outer thin casing of a darker color, slightly tougher than the inner subcutaneous tissue. Due to the somewhat earthy and bitter taste of potato skin, it is often removed before consuming the tuber. In the biocarbon preparation research, dried potato peelings were utilized as a substrate [24].

Residue from black tea (TR) brewing was collected after the extraction of tea essence during the tea infusion process. The descended tea residue, when re-infused with boiling water to create a tea beverage, exhibited no release of tea essence and no alteration in water color. The specific tea variety utilized for this purpose was a classic pure black tea sourced from Ceylon [25]. Upon drying the tea residue, it became exceedingly brittle, devoid of moisture, and retained its original brown color.

The walnut tree, belonging to the walnut family, is a deciduous tree species widely popular in Central and Eastern Europe, as well as across a vast expanse of Asia, the Balkans, the Himalayas, and China. Due to the preference for walnut fruits among birds, particularly those from the crow family, these fruits are often carried over long distances, with birds occasionally burying them in agricultural areas, contributing to their dispersal. Walnut trees boast remarkable longevity, capable of bearing fruit for up to 500 years. The walnut fruit comprises several layers, including the outer green pericarp, a soft covering for the woody shell formed internally. The seed, encased in a membranous shell and commonly known as the kernel, is the edible part of the fruit. In the research, two parts of the walnut fruit were employed. The green part (labeled as WGS), upon drying, lost its firmness and color, transforming into a brittle material with shades of brown and black. The other part, the woody shell (WS), exhibited no alteration in physical properties during drying, except for a reduction in weight due to the loss of a minimal amount of moisture [26].

The research also utilized coffee residue derived from green coffee (GC) as another material. Green coffee is characterized by the absence of roasting before being sold, resulting in harder and earthier qualities in the ground green coffee beans. The residue from green coffee is more resilient, and there is no discernible distinction between its appearance before and after use.

**Figure 2:**
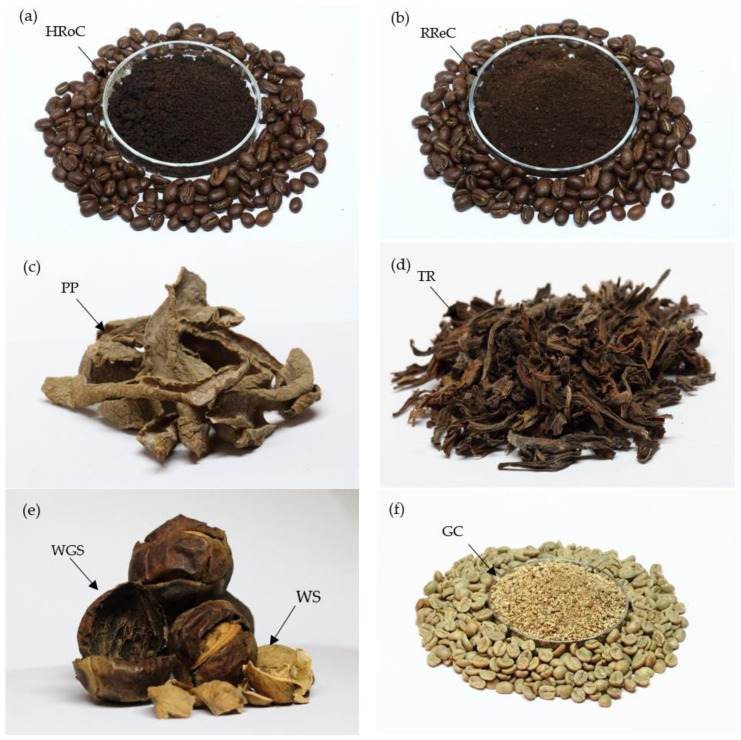
The collected and pre-prepared organic waste, used for the preparation of bioadsorbents: (**a**) heavily roasted coffee residue, (**b**) regular roasted coffee residue (**c**) potato peelings, (**d**) tea residue, (**e**) walnut green shells and walnut shells, (**f**) green coffee residue.

The kinds of dried organic waste described above are depicted in Figure 2a–f. The walnut green shells (WGS) and walnut shells (WS) are presented together as cohesive components (Figure 2e), even though they were subjected to separate processing in subsequent research.

The organic waste underwent a comprehensive cleaning process with tap water, followed by natural drying and further drying in an oven at 105 °C before undergoing carbonization. These dried precursors were subsequently ground into a powder form with dimensions in the range of a few millimeters.

### 2.2. Characterization of Materials 

#### Elemental Analysis of the Samples

The chemical analysis of the organic waste involved both direct and indirect methods. Properties such as moisture content, carbon, hydrogen, nitrogen, and sulfur content were determined. Moisture content in the samples was assessed using the weight-dryer method with a laboratory dryer (SLW 11 with forced air circulation), following the PN-EN ISO 18134-1 standard [27]. 

**Figure 3:**
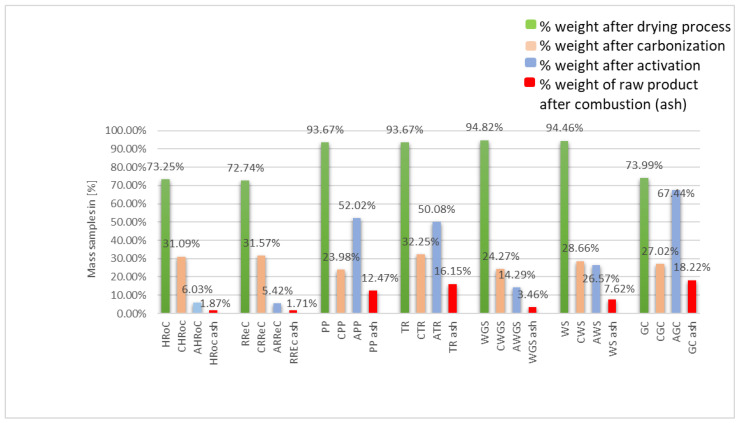
Residual weight of samples by percent at each stage of their processing.

Figure 3 illustrates the mass losses observed at each stage of bioadsorbent preparation. Notably, in the case of coffee residue, there is a noticeable weight reduction during the drying process. This is attributed to the small, dense grain size and the hygroscopic nature of coffee residue, making exclusive air drying impractical. The greater the mass loss during carbonization and activation, the higher the specific surface area of the resulting bioadsorbents. In the case of APP, ATR, and AGC, the mass after activation increased. The final ash weight (above 10%) may indicate poor rinsing of the adsorbent from the activator.

The elemental composition of organic waste, i.e., the determination of the content of carbon, hydrogen, nitrogen, and sulfur, was performed using the LECO Truspec CHNS analyzer. The tests were carried out for samples in a dry state, that were ground into particles less than 0.2 mm in size, in accordance with the requirements of the standard PN-EN ISO 16948:2015-07 [28]. Oxygen was calculated by difference.

Table 3 is a summary of the obtained analysis results for the organic waste used. All materials are characterized by high carbon content and sulfur content. These features are demonstrated by properly selected materials for obtaining carbon materials [29]. The elemental composition of the organic municipal waste subjected to the tests corresponds to the range given in the literature [30,31,32].

## 3. Results and Discussions

### 3.1. Synthesis Bioadsorbents

**Figure 4:**
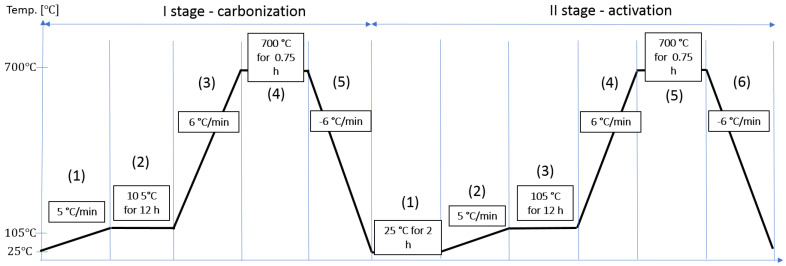
Procedure for the synthesis of bioadsorbents by modifying organic waste.

The modification of the organic waste occurred through two stages: (I) carbonization and (II) activation. Stage I comprises 5 steps: (1) heating the organic waste at the rate of 5 °C/min up to 105 °C, (2) a 12 h period of soaking in a vacuum dryer at 105 °C, (3) heating the samples at the rate of 6 °C/min up to 700 °C in a muffle furnace in a nitrogen gas atmosphere, (4) conducting carbonization at a temperature of 700 °C for 0.75 h, and (5) cooling the sample to 25 °C with the rate of 6 °C/min. Stage II—activation, involves 6 steps: (1) saturating the sample for 2 h with a pre-prepared KOH solution (1 mol), where the solution-to-sample ratio was 2:1, (2) heating samples from 25 to 105 °C in an oven, (3) drying the samples for 12 h in a vacuum dryer at 105 °C, (4) heating the samples at the rate of 6 °C/min up to 700 °C in a muffle furnace in a nitrogen gas atmosphere, (5) activating at a temperature of 700 °C for 0.75 h at a heating rate of 3 °C/min, and (6) cooling the sample to 25 °C (Figure 4). 

The solid bioadsorbents obtained from the activation were washed with distilled water until achieving a neutral pH. The resulting bioadsorbents were then dried at 120 °C for 12 h.

### 3.2. Characterization of Materials

The LECO TruSpec CHN/S analyzer was employed to assess the C, H, N, S, and O contents in the samples following carbonization and activation, as described for organic waste in Section 2.1. The samples’ material underwent degassing at 105 °C for 12 h to eliminate moisture or volatile impurities. Nitrogen adsorption isotherms at −196 °C were determined, covering relative pressures (p/p_o_) from 0 to 0.98 across a broad range of pores. Due to the cryogenic temperature, N_2_ diffusion was restricted in ultra-micropores (<0.7 nm). The surface area of the samples was computed using the Brunauer-Emmett-Teller (BET) equations, and the total pore volumes were calculated based on the adsorbed nitrogen amount at a relative pressure p/p_o_.

The morphology of the materials was investigated using a Carl Zeiss Microscopy GmbH scanning electron microscope (SEM model EVO 15). A vacuum in the range of 0.001–133 Pa and a magnification range of 5×–1,000,000× were required during the microscope chamber examination.

Thermogravimetric analysis (TGA) was conducted on a Mettler TGA/SDTA 851e thermobalance. The samples were initially purged with nitrogen and heated from 25 °C to 1000 °C at a constant heating rate of 10 °C/min.

XRD analysis was carried out to compare organic biomass samples with biomass samples post-carbonization and activation. Measurements were collected using copper radiation (Kα1 = 0.154056 nm) in the 15–60 range on a 2θ scale. Scintillation counter angular speed was set at 0.0010/min, and the patterns were analyzed with Philips X”Pert, utilizing the X’Celerator Scientific detector.

The FTIR spectra of the activated organic municipal waste were recorded at room temperature using a Nicolet 6700 FT-IR spectrometer with the KBr pellet technique [33].

#### 3.2.1. Characterization of Chemical Composition 

Table 4 provides a summarized overview of the analysis results obtained for the various kinds of carbonized organic waste. Materials derived from walnut shells consist of almost 93% carbon. Coffee bean materials (CHRoC and CRReC) exhibit comparable high carbon content, ranging from 66% to 77%. Tea residue (CTR) shows approximately 72% carbon, while potato peelings (CPP) have around 61%. The lowest carbon content is found in walnut green shells (CWGS), accounting for less than 41%. A notable disparity in oxygen content following the carbonization process stems from the loss of the oxide group during the heating stage. This loss of elemental oxygen is attributed to the volatilization of materials, particularly CO and CO_2_ [34].

Table 5 offers a concise overview of the analysis results derived from the activated organic waste (bioadsorbents). In these materials, a modest rise in carbon content is observed, accompanied by a more pronounced reduction in oxygen content. The nitrogen content undergoes essentially insignificant changes. The observable decrease in oxygen-combining compounds indicates further volatilization from the material. Meanwhile, the nearly unaltered nitrogen content during the activation process suggests the incorporation of nitrogen into the aromatic structure rather than in the functional groups on the layer edges [35].

#### 3.2.2. BET Analysis 

Table 6 includes the designated textural parameters and a brief explanation for the determined samples.

The conducted N_2_ adsorption isotherms helped to determine the texture parameters as total pore volume (V_p_), pore size (S_BET_), micropore size (W_o_), and average micropore width (L_o_) summarize Table 6. These analysis results enable a comparison of changes occurring in the samples of organic waste after the carbonization and activation processes. The texture parameters estimated from N_2_ adsorption exhibit similar trends. The porosity develops in each material after the carbonization process and is particularly accentuated after the activation process. Pores within the range of 0.86–1.36 nm were achieved. The surface area increased from <1 m^2^/g for organic waste to 293 ÷ 1604 m^2^/g for activated bioadsorbents. The highest specific surface areas were attained for the activated potato peelings (1604 m^2^/g for APP) and roasted coffee residue (1580 m^2^/g for AHRoC). A slightly smaller pore surface was obtained for the activated sample of green walnut shells (AWGS—1376 m^2^/g). The same pattern applies to the volume of micropores, with the largest area found in the activated roasted coffee residue (AHRoC-0.5 cm^3^/g) and the activated potato peelings (0.32 cm^3^/g for APP). However, in the case of the activated roasted coffee residue (AHRoC), the micropores were the smallest, with an average size of 0.96 nm. The accumulated electric charge, resulting from a large number of ions, caused the sample to adhere to the vessel walls, leading to difficulties in transferring the correct amount of sample and conducting measurements. Lack of parameters for biomass samples from walnut green shells (WGS) and green coffee residue (GC) arose from a poorly developed porous surface, rendering some parameter analyses impossible. The calculated beta area confirms such narrow porosity. The W_o_ and L_o_ values fall within the typical limits described by other authors, affirming the acquisition of microporous bioadsorbents [38,39].

Bioadsorbents obtained by other researchers have demonstrated comparable or inferior structural properties. For instance, Kim et al. achieved a biochar from coffee grounds through one-stage activation with potassium carbonate, yielding a surface area of 1692 m^2^/g [32]. Travis et al. obtained a bioadsorbent from coffee using two-stage activation of KOH, resulting in a specific surface area of 1624 m^2^/g [40]. Zhang et al. obtained a ZnCl_2_ bioadsorbent from potato peelings with a specific surface area of 1604 m^2^/g [41]. For comparison, specific surfaces of other bioadsorbents reported in scientific articles are summarized in Table 7.

**Figure 5:**
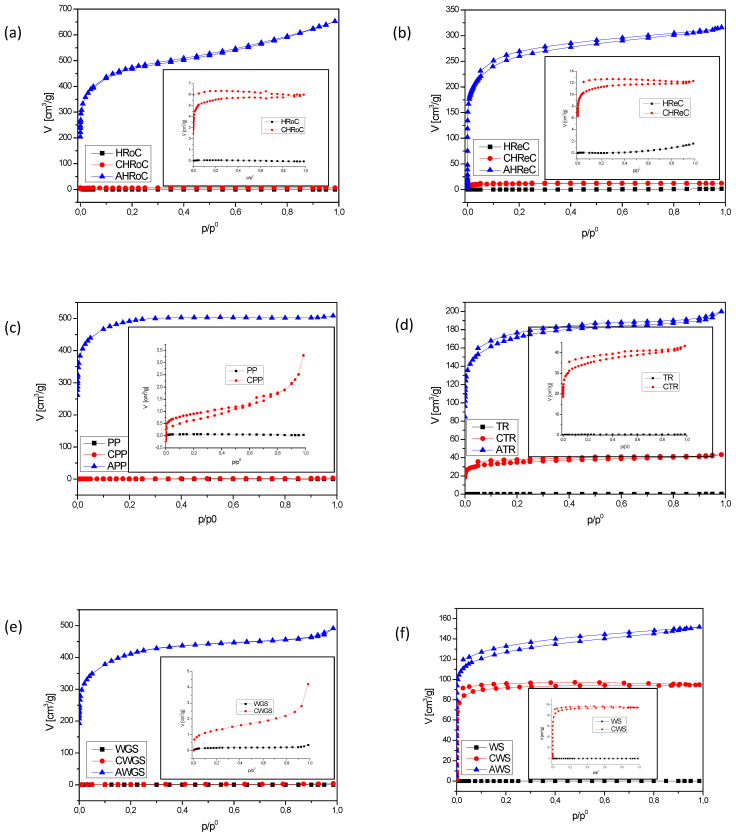

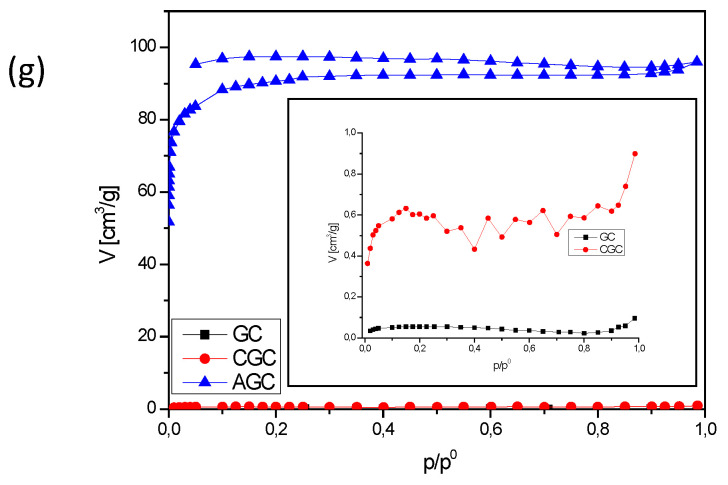
N_2_ adsorption isotherms at −196 °C of samples of organic waste after the carbonization process and after the activation process with (**a**) heavily roasted coffee residue, (**b**) regular roasted coffee residue, (**c**) potato peelings, (**d**) tea residue, (**e**) walnut green shells, (**f**) walnut shells, (**g**) green coffee residue.

Figure 5a–g display the N_2_ adsorption and desorption isotherms for the samples. All activated samples (AHRoC, ARReC, APP, ATR, AWGS, AWS, and AGC) exhibit the International Union of Pure and Applied Chemistry (IUPAC) type I isotherm, indicating that the majority of N_2_ is adsorbed at low pressures without the presence of hysteresis loops. This type I isotherm suggests that the adsorbent is predominantly composed of micropores [42,43]. With increasing severity in activation conditions (high activation temperature or prolonged activation time), there is a rise in N_2_ adsorption uptake. Notably, the degree of N_2_ adsorption increase is more pronounced when altering the activation temperature rather than the activation time. Nitrogen adsorption–desorption isotherms for organic waste samples are nearly imperceptible, with adsorption being barely visible or entirely absent. Only the regular coffee residue (RReC) sample exhibits minimal adsorption (<2 cm^3^/g) and complete desorption. In the case of carbonized samples, visible adsorption–desorption is observed for the carbonized green walnut shell (CWGS) sample, reaching up to 4.5 cm^3^/g. The carbonized walnut shell sample (CWS) displays high adsorption, up to 100 cm^3^/g, but desorption is limited to 90 cm^3^/g. For carbonized roasted and regular coffee residue (CHRoC, CRReC) samples, adsorption reaches up to 6 and 12 cm^3^/g, respectively and desorption is complete. The carbonized tea residue (CTR) sample shows adsorption up to 4 cm^3^/g with complete desorption.

In the case of the carbonized potato peeling (CPP) sample, adsorption reaches up to 3 cm^3^/g with nearly complete desorption. However, for the carbonized green coffee residue (CGC) sample, the adsorptio n–desorption data is unreliable.

**Figure 6:**
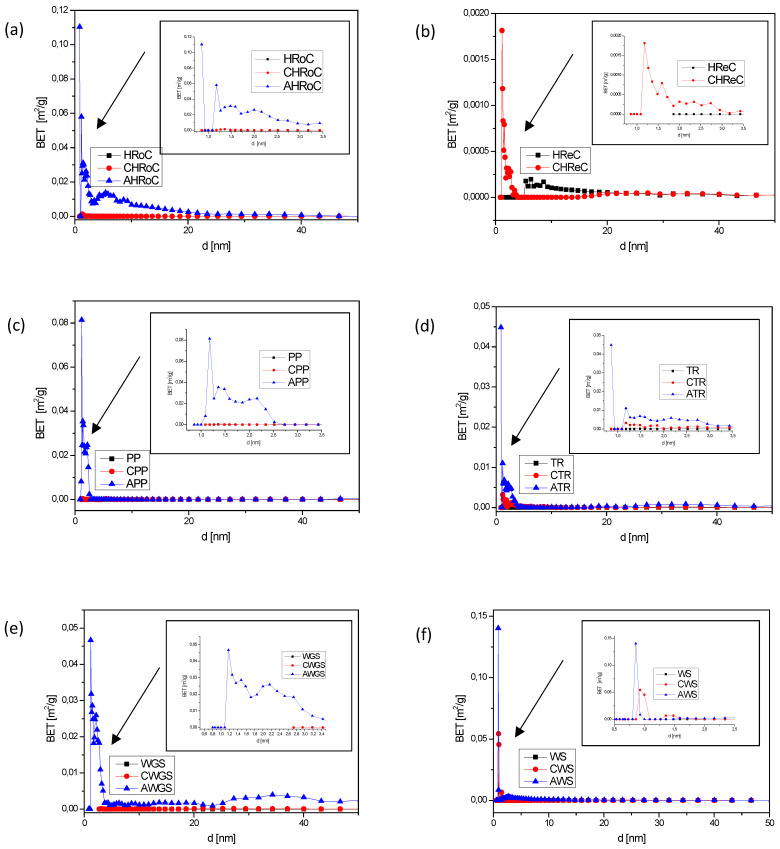

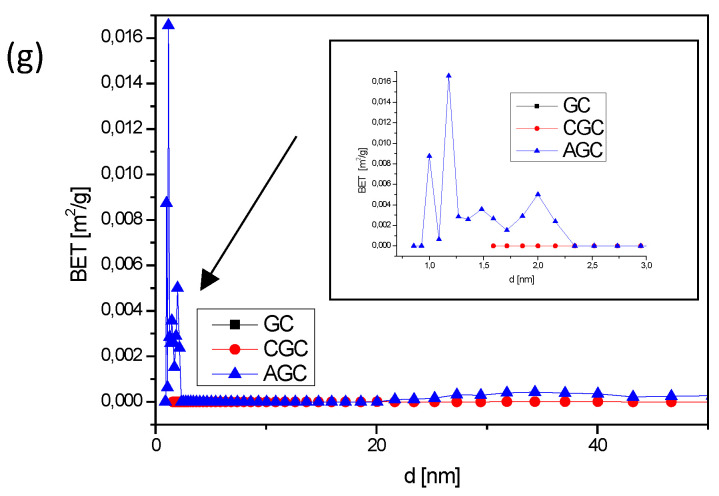
Pore size distributions of materials with (**a**) heavily roasted coffee residue, (**b**) regular roasted coffee residue, (**c**) potato peelings, (**d**) tea residue, (**e**) walnut green shells, (**f**) walnut shells, (**g**) green coffee residue.

Pore size distribution analysis was conducted to examine the textural properties of the samples. As depicted in Figure 6., the pore size distributions for non-activated samples after carbonization are similar, with minimal variations within the margin of error. Generally, these samples exhibit small porosity, which is negligible when compared to the activated samples. The smallest micropores, predominantly below 2 nm and in the highest concentration, are observed in the sample of activated roasted coffee (AHRoC). The absence of a curve for the lightly activated roasted coffee sample is attributed to challenges in working with the sample, which experiences significant electrification. Despite being already carbonized, the sample of regular coffee (CRReC) demonstrates a notable adsorption potential and a substantial number of micropores exceeding 2 nm. In the activated potato peeling sample (APP), the micropore peak is at 1.2 nm, while for the activated tea sample (ATG), it is at 0.8 nm, similar to the activated walnut shell sample (AWS) but with a higher concentration. The activated green walnut shell sample (AWGS) exhibits a broad range of micropores from 1.2 nm to 3.4 nm. Regarding microporosity, the samples of activated roasted coffee display the greatest potential, featuring a wide range of micropores and the highest concentration, surpassing the most developed microporosity observed in the sample.

#### 3.2.3. SEM

**Figure 7:**
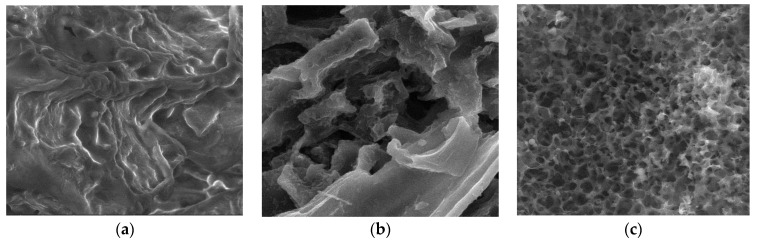
SEM images of (**a**) HRoC, (**b**) CHRoC, (**c**) AHRoC.

**Figure 8:**
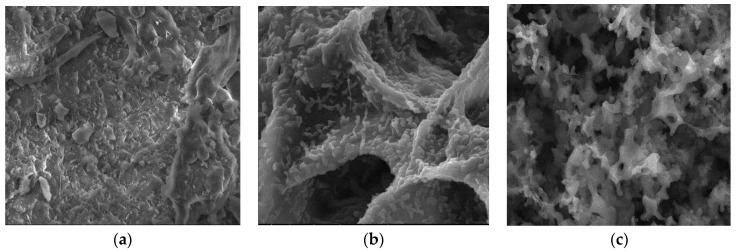
SEM images of (**a**) RReC, (**b**) CRReC, (**c**) ARReC.

**Figure 9:**
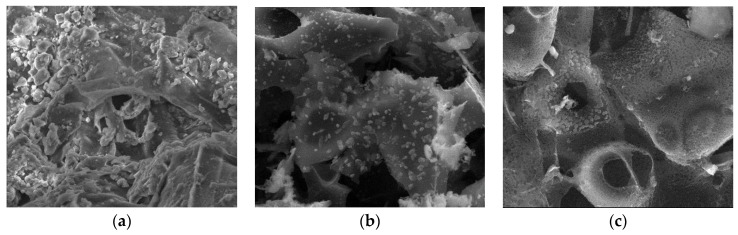
SEM images of (**a**) PP, (**b**) CPP, (**c**) APP.

**Figure 10:**
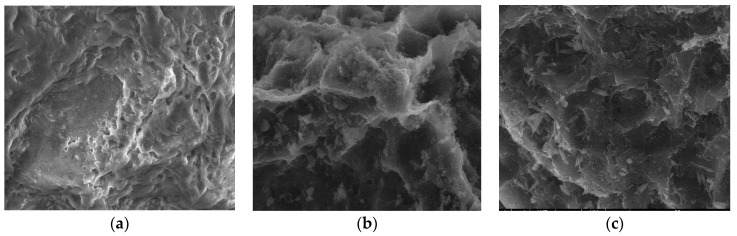
SEM images of (**a**) TR, (**b**) CTR, (**c**) ATR.

**Figure 11:**
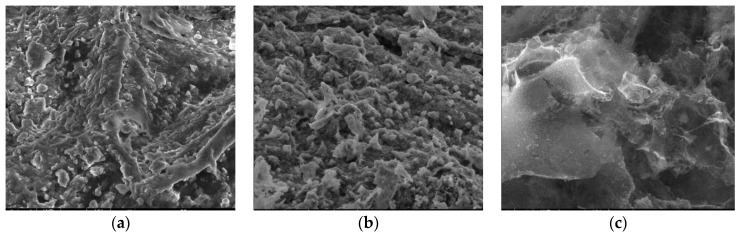
SEM images of (**a**) WGS, (**b**) CWGS, (**c**) AWGS.

**Figure 12:**
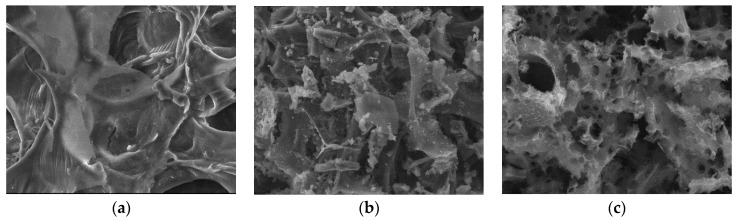
SEM images of (**a**) WS, (**b**) CWS, (**c**) AWS.

**Figure 13:**
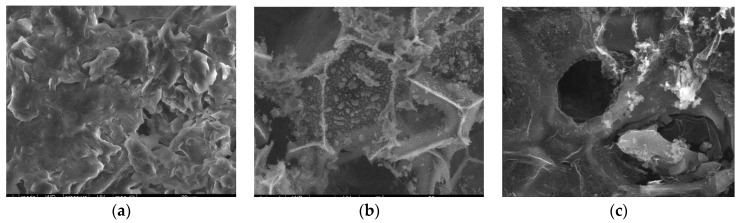
SEM images of (**a**) GC, (**b**) CGC, (**c**) AGC.

Surface morphologies encompass the physical attributes and characteristics of a material’s surface, including its shape, texture, roughness, and topography. To investigate the surface morphology of the materials, scanning electron microscopy (SEM) was employed [44]. SEM images were utilized to illustrate alterations in the internal structure of the samples at key stages of sample preparation [45]. Figure 7a, Figure 8a, Figure 9a, Figure 10a, Figure 11a, Figure 12a and Figure 13a present SEM images of organic waste samples HRoC, RReC, PP, TG, WGS, WS, and GC, while Figure 7b, Figure 8b, Figure 9b, Figure 10b, Figure 11b, Figure 12b and Figure 13b display carbonized organic waste samples CHRoC, CRReC, CPP, CTG, CWGS, CWS, and CGC. Activated organic waste (bioadsorbents) is depicted in Figure 7c, Figure 8c, Figure 9c, Figure 10c, Figure 11c, Figure 12c and Figure 13c—AHRoC, ARReC, APP, ATG, AWGS, AWS, and AGC. The SEM images captured during the examination reveal notable differences in the topography of the external structure of the materials. Even at the carbonization stage, and more prominently after the KOH activation process, substantial porosity becomes evident (images marked with “b”). Following the carbonization stage, macropores are observable in all materials (Figure 7b, Figure 8b, Figure 9b, Figure 10b, Figure 11b, Figure 12b and Figure 13b). In the case of bioadsorbents derived from coffee (AHRoC, ARReC), tea (ATG), and walnut shells (AWS), significant micropores are visible post-activation [46]. Samples of heavily roasted coffee (AHRoC) exhibit a very regular network of micropores after the activation process (Figure 7c). Slightly less regular but still significant microporous structures are observed in the sample of activated regular coffee (ARReC) (Figure 8c). The structure of the activated walnut shell sample (AWS) is noteworthy, featuring a network comprising both macro and micro pores (Figure 12c). The compiled photos of the samples were captured at the same magnification to facilitate better analysis and comparison.

#### 3.2.4. TG Profiles

**Figure 14:**
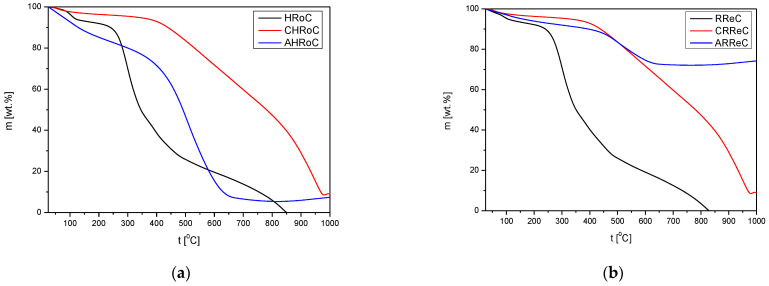

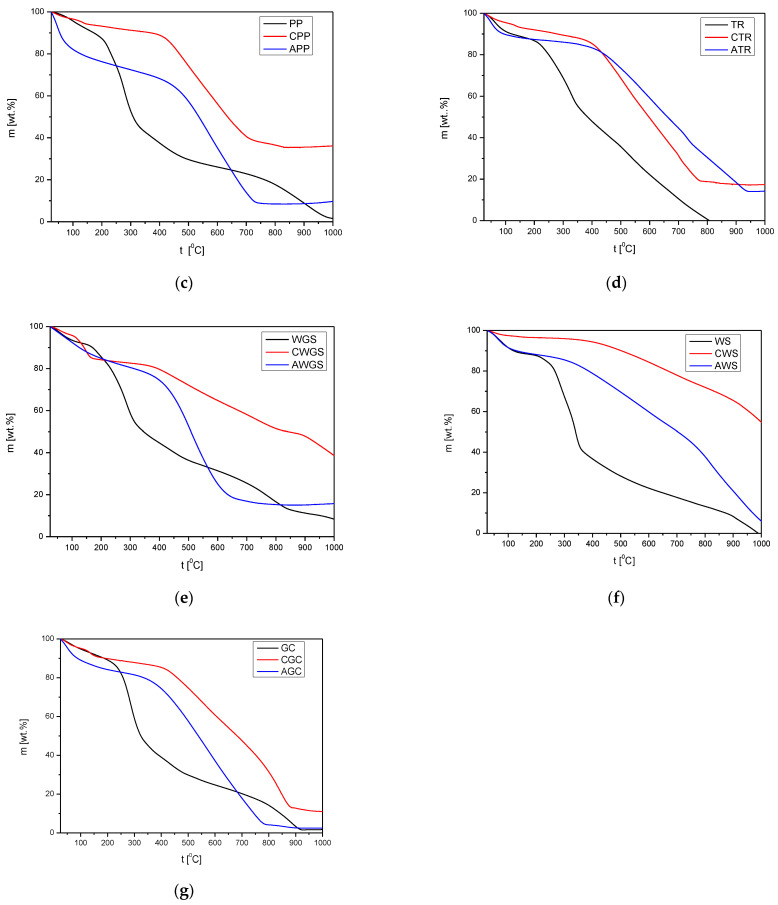
TGA profiles for (**a**) heavily roasted coffee residue, (**b**) regular roasted coffee residue, (**c**) potato peelings, (**d**) tea residue, (**e**) walnut green shells, (**f**) walnut shells, (**g**) green coffee residue.

An analysis of each sample of the materials was carried out to examine the thermal stability of materials at 25–1000 °C in a nitrogen atmosphere. In order to compare the changes occurring in the structures of the compounds in the modification processes, samples from the same kinds of waste are presented in the Figure 14. Common points of mass faults can be seen for dry biomass samples without modification. At 272 °C, hemicellulose degradation occurs, at 331 °C cellulose degradation occurs, and at 377 °C decomposition of lignin occurs [47]. In the case of samples after the carbonization and activation processes, a similarity can be found in the plots with a lower mass loss for the activated samples. The thermal stability of coffee samples after the activation process is reached much earlier, at about 450 °C. The weight loss around 600 °C for the activated coffee samples is attributed to skeletal condensation.

The weight loss curves for the carbonized and activated potato peel sample are very similar (Figure 14c,e,g). Thermal stability is obtained at 650–700 °C, and rapid weight loss occurs between 450 °C and 650 °C.

The course of the curve for the decomposition of the carbonized and activated tea samples is very similar, except that the thermal stability for the carbonized sample is reached at 750 °C, and for the activated one, it is reached at 900 °C.

The course of the curve for the decomposition of the activated green walnut shell sample is similar to that of the activated potato peeling sample. Stability is reached at 650 °C, and the last peak weight loss lasts from 450 °C to 650 °C. For the carbonized sample, weight loss begins at 450 °C with no clear end.

The course of the curve for the decomposition of the carbonized and activated walnut shell samples is similar—no thermal stability.

The course of the curve for the decomposition of the carbonized and activated green coffee samples is very similar, except that the thermal stability for the activated sample is reached at 750 °C, and for the carbonized one, it is reached at 850 °C.

#### 3.2.5. XRD Analysis 

**Figure 15:**
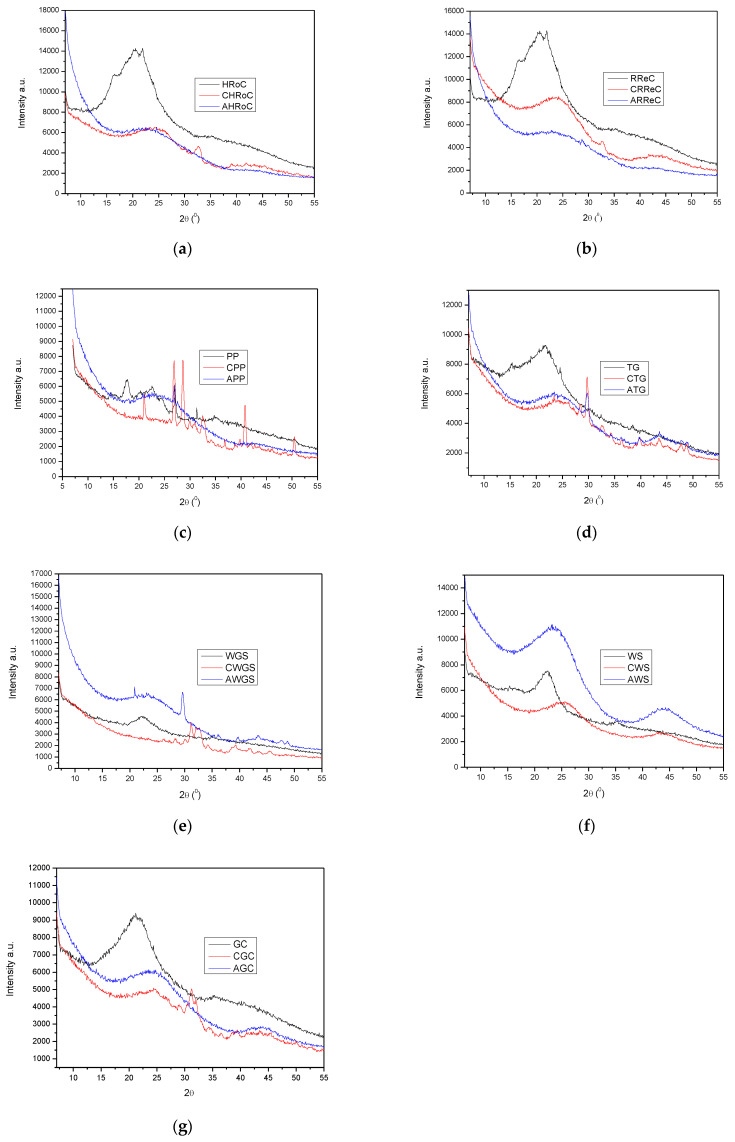
XRD patterns of the samples with (**a**) heavily roasted coffee residue, (**b**) regular roasted coffee residue, (**c**) potato peelings, (**d**) tea residue, (**e**) walnut green shells, (**f**) walnut shells, (**g**) green coffee residue.

The XRD patterns of the samples are shown in Figure 15. The obtained results were compared with those reported in the International Center for Diffraction Data (ICDD). Every sample shows broad peaks at about 2θ = 24° and 44°. These peaks can be related to the typical characteristics of carbon [48]. Along with the modifications carried out, such as carbonization and then activation of organic waste, the intensity of the peaks decreases and smoothing occurs. This is due to the disorder of the carbon. Mallesh et al. showed similar changes in the XRD analysis of an adsorbent they prepared based on the shells of a local nut activated with sodium hydroxide [49]. The broad peak at 24° is associated with the graphite system and represents a highly disordered crystalline structure consisting of both aliphatic side chains and an amorphous carbon structure. Compared to the broad diffuse peak in amorphous carbon, the graphite band at 44° indicates the presence of an aromatic structure. The slight sharp peaks at 2θ = 32.3° can be attributed to the modifications caused by the activation with potassium hydroxide. Reflections like others at 2θ = 31.2, 44.6, 50.1° are characteristic of samples activated with potassium hydroxide. Lahijani et al., who activated the obtained biochar with NaNO_3_, also showed peaks at the above-mentioned degrees [50].

#### 3.2.6. FT-IR Analysis

**Figure 16:**
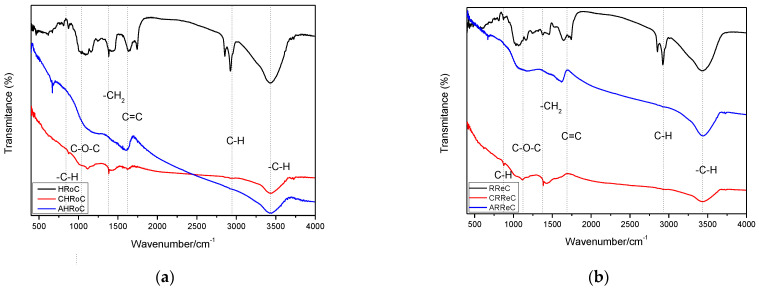

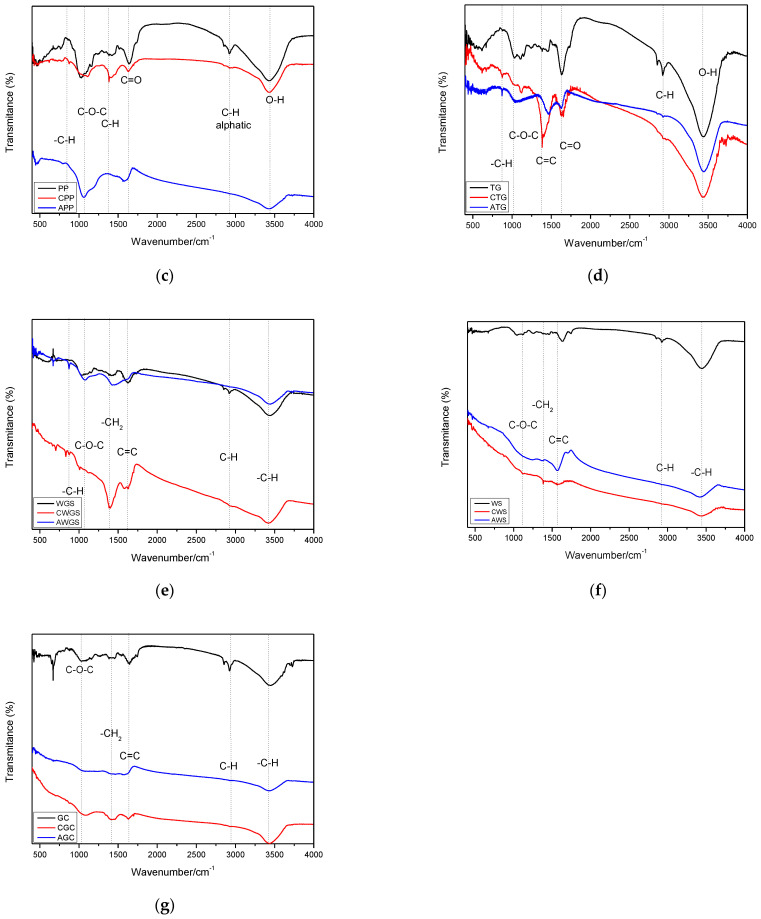
FTIR spectrum of organic waste and the products received with (**a**) heavily roasted coffee residue, (**b**) regular roasted coffee residue, (**c**) potato peelings, (**d**) tea residue, (**e**) walnut green shells, (**f**) walnut shells, (**g**) green coffee residue.

Figure 16a–g show the FTIR spectra samples of organic waste carried out on biomass and samples after modification of organic waste (after process carbonization and activation). Each of the spectra has several characteristic bands in close positions. The wide transmission bandwidth at 3315 cm^−1^ is attributed to –O-H stretching. Another peak in the spectra around 2900 cm^−1^ is the stretching bond –C-H [35]. The peak at 1700 cm^2^ corresponds to the C=C stretching skeleton and 1024 cm^−1^ corresponds to asymmetric =C–O–C stretching vibrations. The peak at 750 cm^−1^ visible in some spectra is the –C-H bond [51]. This bond is visible for samples (ARoC, AReC, ATG, AWGS) that are enhanced by increasing the mass ratio of KOH to biomass. It indicates that the activation of KOH promotes the graphitization of activated carbons [29].

All spectra show that in pure biomass samples there are bands before 3000 cm^−1^ corresponding to aliphatic groups -C-H. The spectra of pure biomass are characterized by greater variability, and with a greater number of peaks in each spectrum, along with the introduced modifications, the spectrum becomes smoother.

Table 8 summarizes the activation conditions and the most important textural parameters for the samples of this article and those obtained for samples from similar precursors. The results obtained for specific surface area (S_BET_), pore volume (V_p_), micropore volume (W_o_), and average micropore width (Lo) are better for the samples in this article except for the sample of activated walnut shells. Not all parameters could be compared nor could all precursors. There is a lack of data in the literature that would allow a complete comparison of these parameters.

It should be noted that the values given in this work are higher than those available in the literature for analogous carbons. Only the bioadsorbents obtained by Boudrahem and others from coffee waste under the same activation conditions have a slightly higher specific surface area but a smaller pore volume. 

The results also prove that the potassium hydroxide used is a better activator than other substances. None of the other activators used, such as phosphoric acid(V), zinc chloride, and others, allowed for such a strong development of the porous structure of bioadsorbents.

The average micropore values indicate high crystallization of the samples. This proves the potential use of these bioadsorbents for pollutant capture purposes.

## 4. Conclusions

The literature currently lacks information on changes in the specific surface area of organic waste before modifying the structure of the material. Researchers focus on examining already obtained bioadsorbents, without comparing their properties before modification. Moreover, articles often focus on one type of waste, which makes it difficult to compare the properties of waste obtained under similar conditions.

This article carried out research at all stages of structural modifications. Material tests were carried out both for non-activated materials, after the carbonization process and after the activation process.

As a result of the work carried out to carbonize and activate seven different types of waste biomass, the most effective adsorbent turned out to be a bioadsorbent derived from roasted coffee residues. After activation, this adsorbent had a well-developed specific surface area of 1580 m^2^/g (in relation to the range of 593–1624 m^2^/g from the literature), a visible structure and a pore volume of 0.84 cm^2^/g. A bioadsorbent from potato peels with a surface area of 1604 m^2^/g and a pore volume of 0.65 cm^2^/g turned out to be an effective sorbent.

The worst starting material for obtaining a bioadsorbent was walnut shell, as evidenced by the surface area of 416 m^2^/g.

Analyzes of raw biomass materials as well as the intermediate product before the final preparation of the bioadsorbent also showed: a large weight loss and a change in structure towards porosity in the carbonization phase. Only chemical activation of KOH resulted in a significant increase in the surface area, which confirms that chemical activation is an effective activation method.

## Figures and Tables

**Table 1:** Comparison of bioadsorbents’ properties, which depend on raw material and production procedure.

Raw Material	Coconut Shells		Rice Husks		Pomegranate Peels	Carrot Peels	Coffee Residue	
annual production	>25 Mtons (Southeast Asia and the Asia-Pacific region) [11]		~700 Mtons (global production) [12,13]		~30 Mtons (global production) [14]		~15 Mtons (global production) [15]	
activation process	two-stage activation process involving urea and KOH [16]	single-stage physical activation process using carbon dioxide	two-stage activation process involving KOH and chitosan	single-stage activation the organic waste with KOH	one-stage activation with potassium hydroxide	activation with the same chemical compound	one-stage physical	one-stage chemical activation with KOH
adsorbent specific properties achieved	1687 m^2^/g 83% of C	1357 m^2^/g	1500 m^2^/g, 82% of C	2695 m^2^/g	2144 m^2^/g	1376 m^2^/g	534 m^2^/g 82% of C	840 m^2^/g
Ref.	[16]	[17]	[18]	[19]	[20]	[21]	[22]	[23]

**Table 2:** Designations of organic waste and products of their modification used in the article.

No.	Organic Waste	Designation	Designation of Organic Waste after Carbonation	Designation of Organic Waste after Activation
1.	Heavily roasted coffee residue	HRoC	CHRoC	AHRoC
2.	Regular roasted coffee residue	RReC	CRReC	ARReC
3.	Potato peelings	PP	CPP	APP
4.	Tea residue	TR	CTR	ATR
5.	Walnut green shells	WGS	CWGS	AWGS
6.	Walnut shells	WS	CWS	AWS
7.	Green coffee residue	GC	CGC	AGC

**Table 3:** Chemical characterization of organic municipal waste.

Sample		Unit	HRoC	RReC	PP	TR	WGS	WS	GC
Element	C	wt.%	44.75	48.59	42.85	43.93	43.94	45.28	38.82
	H	wt.%	5.88	6.31	5.89	5.43	5.38	5.85	5.60
	N	wt.%	2.08	2.32	2.37	2.89	0.77	0.39	1.72
	S	wt.%	0.00	0.01	0.05	0.08	0.01	0.00	0.09
	O	wt.%	47.29	42.77	49.34	49.83	49.90	48.48	53.77

**Table 4:** Chemical composition of various carbonized materials from kinds of organic waste.

Sample		Unit	CHRoC	CRReC	CPP	CTR	CWGS	CWS	WGC
Element	C	wt.%	66.69	77.04	61.20	72.76	40.72	95.58	69.80
	H	wt.%	1.31	1.55	1.22	1.47	1.26	1.39	1.24
	N	wt.%	2.92	3.88	2.23	2.95	0.69	0.58	2.73
	S	wt.%	0.00	0.01	0.01	0.06	0.01	0.00	0.06
	O	wt.%	29.08	17.52	34.34	22.76	57.32	5.45	26.17

**Table 5:** Chemical composition of various bioadsorbents obtained from kinds of organic waste.

Sample		Unit	AHRoC	ARReC	APP	ATR	AWGS	AWS	AGC
Element	C	wt.%	68.89	79.90	63.44	72.59	45.56	93.52	76.41
	H	wt.%	1.84	1.78	3.13	2.04	2.18	2.18	1.59
	N	wt.%	5.36	6.23	2.40	3.82	3.11	3.38	2.89
	S	wt.%	0.00	0.00	0.00	0.01	0.00	0.00	0.02
	O	wt.%	23.91	12.09	31.03	21.54	49.15	0.92	19.09

**Table 6:** Textural parameters determined from the adsorption isotherms of the studied samples.

Textural Parameters		
Total pore volume	V_p_	Amount of N_2_ adsorbed at a relative pressure of 0.99
Surface area	S_BET_	Brunauer-Emmett-Teller equation [32]
Micropore volume	W_0_	N_2_ isotherms: Dubinin-Radushkevich (DR) equation assuming a density of the adsorbed phase of 0.808 cm^3^ g^−1^ and a cross sectional area of 0.162 nm^2^ [36]
Average micropore width	L_0_	Stoeckli-Ballerini equation [37]

**Table 7:** Structural properties of samples.

Sample	N_2_ Adsorption (at −196 ° C)			
	S_BET_	V_p_	W_0_	L_0_
	m^2^/g	cm^3^/g	cm^3^/g	nm
HRoC	0.21	0.00006	n/a	1.54
CHRoC	18	0.008	0.004	1.36
AHRoC	1580	0.84	0.5	0.96
RReC	0.04	0.0024	n/a	n/a
CRReC	37	0.02	0.01	1.6
ARReC	863	n/a	n/a	n/a
PP	0.21	0.00005	0.00003	1.36
CPP	3.17	0.004	0.001	1.36
APP	1604	0.65	0.32	1.36
TR	0.52	0.0004	0.0002	1.60
CTR	115	0.05	0.02	1.27
ATR	564	0.25	0.12	0.86
WGS	0.62	0.00	n/a	n/a
CWGS	4.55	0.00	0.00	3.07
AWGS	1376	0.64	0.34	1.21
WS	0.17	0.0002	0.0001	1.6
CWS	289	0.12	0.12	0.93
AWS	416	0.20	0.18	0.86
GC	n/a	n/a	n/a	n/a
CGC	0.18	0.00013	0.00	n/a
AGC	293	0.12	0.05	1.18

**Table 8:** Comparison of the activation parameters and the most important textural parameters for the samples in this article with the literature data.

Precursor	Activation Conditions		Textural Parameters				
	Activation Precursor	T [°C]	S_BET_ [m^2^/g]	V_p_ [cm^3^/g]	W_o_ [cm^3^/g]	L_o_ [nm]	Ref.
Coffee grounds	H_3_PO_4_	450	925	0.718	n/a	n/a	[52]
Coffee grounds	CO_2_	700	593	0.24	0.24	0.8	[23]
Coffee grounds	Steam	800	981.12	1.03	n/a	4.19	[53]
Coffee residue	H_3_PO_4_	600	1003	0.618	n/a	n/a	[54]
Coffee residue	KOH	700	1624	0.662	n/a	n/a	[35]
Coffee residue	KOH	700	1580	0.84	0.5	0.96	This study
Waste potato	ZnCl_2_	600	1357	1.065	n/a	n/a	[42]
Waste potato	KOH	700	1383	0.736	0.234	n/a	[55]
Waste potato	KOH	700	1604	0.65	0.32	1.36	This study
Tea residue	C_2_H_3_O_2_K	800	820	0.219	n/a	n/a	[56]
Tea residue	KOH	500	256.45	n/a	n/a	n/a	[57]
Tea residue	KOH	700	564	0.25	0.12	0.86	This study
Walnut shell	H_3_PO_4_	500	789	0.304	n/a	n/a	[58]
Walnut shell	KOH	700	416	0.2	0.18	0.86	This study

## Data Availability

Data are contained within the article.
